# Flow Cytometry Confirmation Post Newborn Screening for SCID in England

**DOI:** 10.3390/ijns8010001

**Published:** 2021-12-23

**Authors:** Kimberly C. Gilmour

**Affiliations:** Immunology Laboratory, Camelia Botnar Laboratories, Hospital for Children NHS Foundation Trust, Great Ormond Street, London WC1N 3JH, UK; kimberly.gilmour@gosh.nhs.uk

**Keywords:** severe combined immunodeficiency, TREC, flow cytometry, T cells

## Abstract

An evaluation program for newborn screening for Severe Combined Immunodeficiency began in England in September 2021 based on TREC analysis. Flow cytometry is being used as the follow up diagnostic test for patients with low/absent TRECS. The immunology laboratories have established a protocol and values for diagnosing SCID, other T lymphopenias and identifying healthy babies. This commentary describes the flow cytometry approach used in England to define SCID, T lymphopenia and normal infants after a low TREC result. It provides background to the flow cytometry assays being used and discusses the need to monitor and potentially change these values over time.

## 1. Introduction

Infants with severe combined immunodeficiency (SCID) usually present in the first year of life with recurrent, opportunistic infections as well as failure to thrive. Their immunology is characterised as lacking naïve T cells. Without haematopoietic stem cell transplant or gene therapy, most babies die in the first year of life. Since outcomes for SCID are significantly improved if diagnosed at birth [1], a number of countries have introduced screening programs for SCID based on measuring the number of T cell receptor excision circles (TRECS) that are present in the blood spot on a Guthrie card. Low/absent TRECS suggest that the infant may have SCID. However, measurement of TRECS is a screening test that identifies babies with SCID as well as other conditions, including Di George syndrome, CHARGE, idiopathic T lymphopenia and a number of normal infants will be referred for further investigation [2]. To separate infants with SCID and other T lymphopenia conditions from normal infants, flow cytometry (FACs) is used to enumerate lymphocytes, specifically naïve T cells. FACs is a well validated methodology that uses fluorescently labelled antibodies to bind to cell makers. These are run as a single cell stream through a laser whose light is reflected/refracted depending on the fluorescent antibodies bound to the cell as well as cell size and granularity. The resulting changes in light patterns are detected. These can be visualised and quantified by computer software enabling characterisation and enumeration of the cells.

## 2. Flow Cytometry for SCID in England

**Figure 1:**
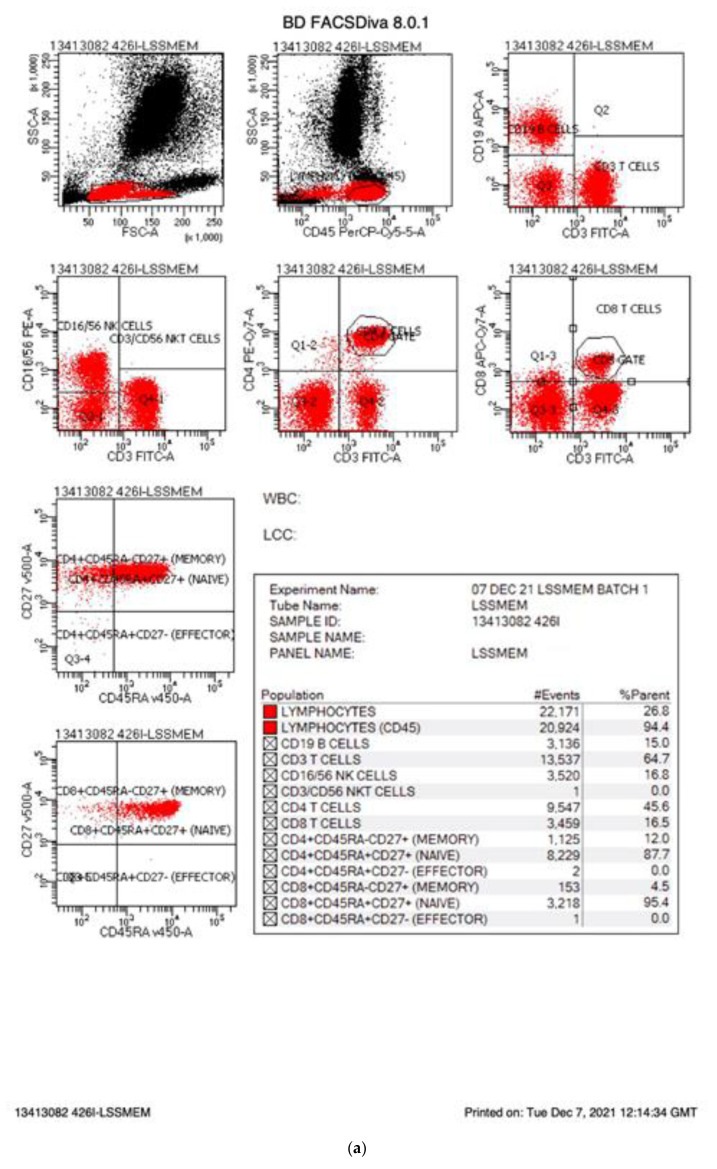

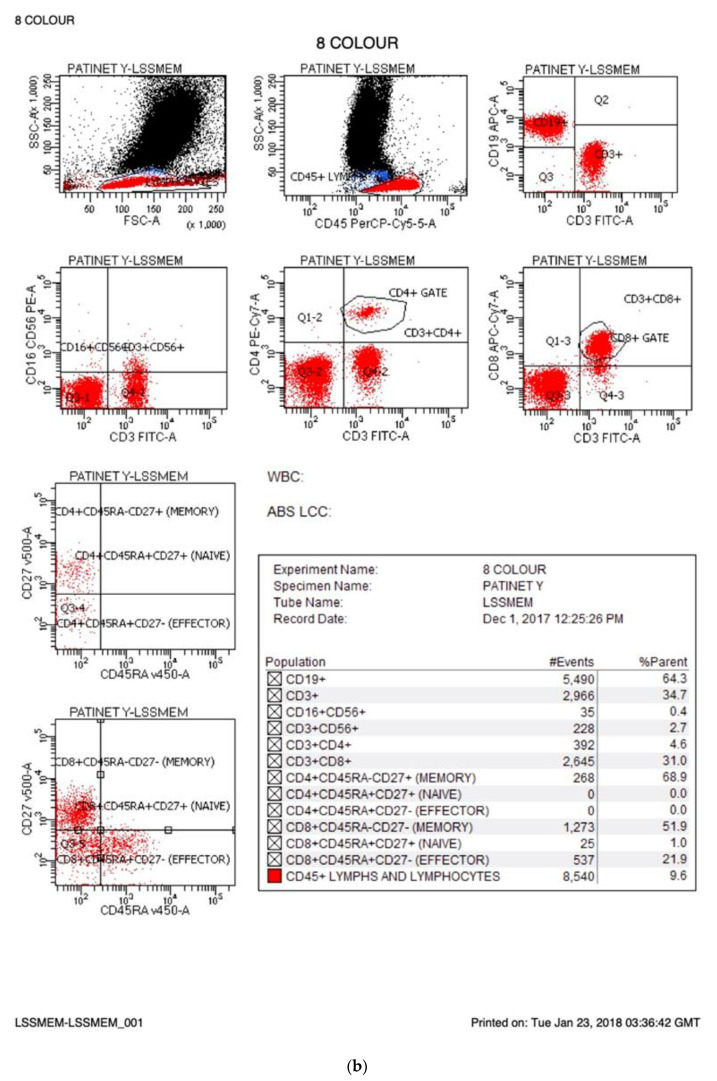
(**a**) Normal FACs plots. The FACs plots from a normal infant are shown. The cells markers for each plot are listed on the X and Y axis and the percentages for each cell population are provided in the statistics box. The CD makers and the cells they identify are summarised in Table 1. (**b**) FACs plots from an infant affected by SCID.

On 6 September 2021, England started an evaluation program for newborn screening for SCID. Two methods are being evaluated: The Perkin Elmer EnLite kit using a cut off of 20 TRECs and the Spot-It Kit from Immuno IVD using a cut off of 8 TRECS. In England, babies identified as having low TRECs are referred to their regional immunology centre where 0.5 mL EDTA blood is collected from the infant, transported to an immunology laboratory where lymphocyte subsets are measured with results returned to the family the same day, often within 2 h of collection. This rapid turnaround has been designed to minimise anxiety for the family and ensure optimal treatment for those patients diagnosed with SCID or other T cell lymphopenias. Although follow up to abnormal TREC analysis varies [3], England measures the percentage and absolute counts of B cells, T cells including CD4 and CD8s T cells, and NK cells. These are assayed by measuring the percentage of these cells as a proportion of lymphocytes and multiplying the percentage by the absolute lymphocyte count or calculating the absolute counts by measuring a fixed number of beads in a fixed volume and calculating the absolute counts based on that. The percentage of naïve/memory T cells is reported as the percentage of CD4 or CD8 T cells after gating on CD4 or CD8 T cells, respectively. MHC II expression on B cells or monocytes (antigen-presenting cells) should be 100%. To accommodate suboptimal samples handling, a figure greater than 90% has been deemed normal. Table 1 summarises lymphocyte populations, normal ranges as well as abnormal ranges and those values diagnostic of SCID. Patients with less than 25% naive T cells are defined as SCID as well as those lacking (<10%) DR expression (MHC II SCID), while hypomorphic MHC II deficiency is defined as 10–90% DR expression. T lymphopenia patients (abnormal) are defined with an absolute CD3 count of 300–1499 T cells/μL or having less than 70% naïve T cells. These values differ from some other screening programs [4]. Figure 1a shows flow cytometry plots from a normal infant. B, T and NK cells are present in normal percentages and the patient has more than 70% naïve T cells. Figure 1b shows flow cytometry from an infant genetically confirmed to have JAK3 SCID. These plots show that the JAK3 infant had 64% B cells, few NK cells (0.4%) and low T cells (35%) with few CD4 T cells (4.6%). Almost none of the T cells present in the JAK3 infant were of a naive phenotype (0% of CD4s and 1% of CD8s) consistent with a diagnosis of T-B+NK- SCID. To ensure consistency and quality across laboratories, all participating laboratories are UKAS accredited to ISO15189. All the laboratories participate in the UKNEQAS (United Kingdom National External Quality Assessment Scheme) Immunophenotyping scheme. In addition, prior to commencement of NBS for SCID, three blood samples were sent to the participating immunology laboratories and immunophenotyping results were compared across the laboratories, showing equivalent results. The laboratories have supported each other by sharing standard operating procedures and where there are questions, sharing flow cytometry plots for additional views. The goal is to ensure rapid, consistent diagnosis of SCID by all participating laboratories.

## 3. Comparison of English Values with Other SCID Screening Programs

As mentioned above, the English evaluation has chosen values to define SCID as less than 25% naïve T cells as defined by CD45RA+ and CD27+ and abnormal less than 1500 CD3 T cells or less than 70% naïve T cells. Although these are consistent with the Primary Immune Deficiency Treatment Consortium [6], they differ from some other programs. Across the first eleven states in the United States to implement newborn screening for SCID, values were based on T cells number (less 300 T cells/μL was defined as SCID and leaky SCID as between 300 and 1500 T cells) as well as the ability of T cells to proliferate to mitogen (2). In California, SCID has been defined as less than 300 T cells/μL or less than 2% CD45RA+ (naïve) T cells or less than 10% proliferation compared to control values. California uses less than 1500 T cells/μL to identify abnormal T cell numbers so as to detect hypomorphic/leaky SCIDs [4]. Catalonia (Spain) defines SCID as less than 300 T cells/μL and uses CD45RA+ and CD45RO+ (memory T cells) in their analysis, although specific values are not stated [7]. A Clinical Immunology Survey of laboratories providing diagnostic testing following an abnormal TREC result showed that the majority of respondents used FACs as their next test. However, the cell markers, particularly those used to identify naïve T cells varied, although the authors stated the importance of including naïve T cells markers in the analysis [3]. England chose to use CD45RA+CD27+ to define naïve T cells as they show a good correlation with thymic emigrants [8]. Values have been set at a slightly higher level (25% naïve T cells) to minimize the chance of missing an atypical or leaky SCID. In England, patients who are defined as SCID by FACs will be referred to a specialist tertiary center and have either a targeted whole exome panel or whole genome sequencing undertaken. As patients are reviewed and genetic results become available, both the TREC cut off and the immunophenotyping values will be reviewed and changed if required.

## 4. Summary

England has started newborn screening for SCID. Patients with low TRECS are referred for flow cytometry analysis to clarify if they have SCID, another T lymphopenia or are healthy infants. The immunology laboratories have established immunological values for diagnosis of SCID and T lymphopenia, with a common methodology being utilised. These values will be monitored as part of the pilot program for newborn screening for SCID.

## Figures and Tables

**Table 1:** Summary of lymphocyte subsets listing common name, CD/FACs markers, a normal range for infants 1 week to 4 weeks of age, values for defining SCID and abnormal (T lymphopenia).

Lymphocyte Population	FACs/CD Marker	Normal Range for 1–4 Weeks [5] Percentage Absolute Counts (Cells/μL)	Range for SCID Percentage Absolute Counts (Cells/μL)	Range for Abnormal/T Lymphopaenia Percentage Absolute Counts (Cells/μL)
T cells	CD3+	60–85% 2300–7000	<300	<30% T cells < 1500
B cells	CD19+	4–26% 600–1900		
NK	CD16+/56+	3–23% 200–1400		
CD4 T cells	CD3+CD4+	41–68% 1700–3500		
CD8 T cells	CD3+CD8+	9–23% 400–1700		
Naïve T cells	CD45RA+/CD27+_	80–100%	<25%	<70%
MHC II expression on APCs (B cells or monocytes	DR+	100%	<10%	<90%
